# Strengthening health research capacity in sub-Saharan Africa: mapping the 2012–2017 landscape of externally funded international postgraduate training at institutions in the region

**DOI:** 10.1186/s12992-018-0395-0

**Published:** 2018-07-31

**Authors:** Terra Morel, Dermot Maher, Thomas Nyirenda, Ole F. Olesen

**Affiliations:** 1 University of Toronto, Toronto, Canada; 2 The Special Programme for Research and Training in Tropical Diseases (TDR), c/o World Health Organization, Avenue Appia, 1211, 27 Geneva, Switzerland; 3 European & Developing Countries Clinical Trials Partnership, Cape Town, South Africa; 4 European & Developing Countries Clinical Trials Partnership, The Hague, the Netherlands

**Keywords:** Health research, Postgraduate training, Sub-Saharan Africa, External funding, Research capacity

## Abstract

**Background:**

The objective was to guide key stakeholders on future directions of external funding of international postgraduate training (Master’s and PhD) of health research students at institutions in sub-Saharan Africa by mapping the numbers and characteristics of students, the location of institutions, and sources of external support.

A cross-sectional survey of eligible external funding organizations and programmes was conducted in 2017. Information was gathered from funders’ websites or through the assistance of institutional contacts. The information requested included the number of Master’s and PhD grantees supported from January 2012 to June 2017, as well as each grantee’s institution of study, gender, country of origin and research area.

**Results:**

Of 72 organizations contacted, there were 44 responses. Of the 44, 30 funders reported programmes within the inclusion criteria, and 19 funders provided data on relevant programmes. The Wellcome Trust, the International Development Research Centre and the Norwegian Agency for Development Cooperation supported the greatest number of grantees. There was concentrated support for grantees in eastern and southern Africa, countries with developed research capacity, and highly-developed research and training centres. More support was provided for PhD than Master’s degree programmes and for research areas more upstream along the research spectrum. Challenges were identified in recognizing relevant funding organizations and obtaining responses. Information was presented inconsistently across organizations, which were often unable to provide relevant and complete data within the survey timeframe.

**Conclusions:**

External funders should collect, analyse and report data at regular intervals on their support for strengthening postgraduate health research capacity in sub-Saharan Africa. Standardization of this process and development of an online database would not only help to avoid overlap between programmes and promote synergy between funders, but also inform dialogue between external funders and key stakeholders on strategic issues. These issues include how external funders can a) optimise their support for research capacity strengthening to maximise the benefits of research for health and development on an equitable basis, and b) optimise the distribution of support for researchers at different career stages and for research on different parts of the research spectrum to maximise the health benefits of research.

**Electronic supplementary material:**

The online version of this article (10.1186/s12992-018-0395-0) contains supplementary material, which is available to authorized users.

## Background

Building and sustaining research capacity has been championed as a leading strategy to overcome health disparities worldwide [1]. Health researchers develop innovative ideas, technologies and approaches to improve the quality of health care [1]. Deficiency in this human resource, and the resulting inability to develop solutions to critical healthcare challenges, is a primary determinant of poverty in low- and middle-income countries (LMICs) [2]. Among LMICs, nations in sub-Saharan Africa face the greatest health system gaps, further exacerbated by low health research capacity. The region accounts for 10% of the global population and only 1.3% of global health research publications [3, 4]. There is also great disparity in research capacity between countries: ranked by the number of researchers per million inhabitants, South Africa is top (818), while Burundi, the Central African Republic, the Gambia, Lesotho and Zambia are at the bottom (each with less than 50) [5].

A comprehensive approach is needed to strengthen health research capacity in the region [6]. This includes mapping of such activities in the region as a first step in assessing their effectiveness and impact [7]. In support of national efforts to grow and retain a critical mass of health researchers in LMICs, external funders may play an important collaborating role in helping to finance health research capacity strengthening activities in sub-Saharan Africa. Specifically, funders may support individual level approaches that contribute to expansion of a national research workforce with the capacity to develop contextually relevant solutions and advance national health priorities [8]. Given the complexity of the landscape of external funding of health research capacity strengthening, there is a need to promote synergy between funders [9, 10]. Mapping this landscape would enable the identification of areas of complementarity, overlap and deficiency, and guide key stakeholders on future directions of this external support.

External support for students for postgraduate degree training (i.e. Master’s and PhD levels) can contribute to the development of a national research workforce, and strengthen institutional and national research output, and dominates the funding landscape. Whereas externally supported researchers from sub-Saharan Africa have often undertaken postgraduate degrees at institutions in high-income countries, there is now an increasing trend for them to study at institutions in the region. This has the benefit of strengthening capacity at the sub-Saharan African institution in addition to training the individual researcher. To contribute towards a complete picture of all funding for postgraduate training of health researchers from sub-Saharan Africa, we mapped the landscape of externally funded international Master’s and PhD health research training at institutions in sub-Saharan Africa. We surveyed the numbers and characteristics of students, the location of institutions, and sources of external support over a five years’ timeframe (2012–2017).

## Methods

### Mapping process

We mapped the landscape of externally funded international postgraduate health research training of students at the Master’s and PhD level at institutions in countries in sub-Saharan Africa. Table 1 shows the inclusion criteria for mapping. The mapping exercise was undertaken between 1 May and 5 July 2017, thereby establishing a period prevalence of externally funded international postgraduate grantees over a five years’ timeframe (2012–2017).

**Table 1:** Inclusion criteria for mapping

Information collected	Inclusion criteria for mapping
Location of external funder	Outside of grantee’s country of origin
Grantee country of origin	In sub-Saharan Africa
Location of grantee institution of study	In sub-Saharan Africa
Degree type	Master’s or PhD

We generated a list of external funders who supported postgraduates to study health research at institutions in sub-Saharan Africa from three main documentary sources and an internet search. The three documentary sources were: 1) the Royal Tropical Institute’s (KIT) “Mapping of Health Systems Research Institutions in Eastern and Southern Africa” [11], which provided contacts at research institutions in countries in these regions to whom we sent queries asking which, if any, external funders made relevant contributions at their institutions; 2) the National Institute of Health’s (NIH) World RePORT [12], which provided information on relevant programmes and funders; and 3) “Health Research Capacity Strengthening: A UKCDS Mapping” [9, 10], which provided details on which funders support the relevant programmes. In conducting internet searches for relevant scholarship programmes, we used keywords including “scholarships”, “funding”, “sub-Saharan Africa”, “Master’s”, “PhD”, “sub-Saharan African student”, and “health research”. Search engines and databases consulted include Google, Google Scholar, PubMed, Scholars4Dev, TopUniversities, University of Toronto Libraries, Scholarship-Positions, and Opportunities for Africans.

The resulting list of funders that support postgraduate grantees was then checked and confirmed. Information on relevant external funding was obtained directly from a funder’s website if possible. If not, we requested information on funders’ relevant contributions by email. Matters of programme scope were clarified by telephone. Email addresses were identified by key informants at the Special Programme for Research and Training in Tropical Diseases (TDR) and the World Health Organization (WHO), internet searches, journals or reports, or on referral from institutional contacts from the KIT database [11]. Contact with a funder involved an explanation of the mapping exercise and an inquiry into their relevant programming. A request was then sent to the funder for information on the international postgraduate grantees supported at the relevant institution of study, including gender, country of origin and research area (Table 2). The data received was transferred to a standardized analysis template and assembled to form one data set, which was then analysed altogether.

**Table 2:** Summary of information requested from each funder

Funder	Grantee host institution	Grantees
Name	Type of degree training supported by external funder (Master’s and/or PhD)	Number supported at host institution by external funder (Master’s and PhD)
Location (country)	Location (country)	Country of origin
		Gender
		Area of research

### Operational definitions

For the purposes of this mapping, we defined sub-Saharan Africa geographically (see Additional file 1: Appendix A for the list of countries included). External funding (whether from outside or within the African region) implies the flow of funds from a source external to the country of origin of the grantee, i.e., the country of which the individual is a national. International training refers to students studying at an institution in a country other than their country of origin.

We defined institution of study as the institution where an individual is predominately based while doing their degree courses and where the student is mainly funded; this may include a degree granting or non-degree granting research institute (in which case the student obtains their degree from a different institution, sometimes outside of the region). We included programmes that provided full financial support for postgraduates (Master’s or PhD).

Research area comprised nine different categories along the spectrum of types of health research activity: basic science, research and development (R&D), implementation, social science, health policy, epidemiology, health systems research, health economics and health technology. Clinical research was classified as R&D.

## Results

### Funders, organizations and programmes

**Fig. 1:**
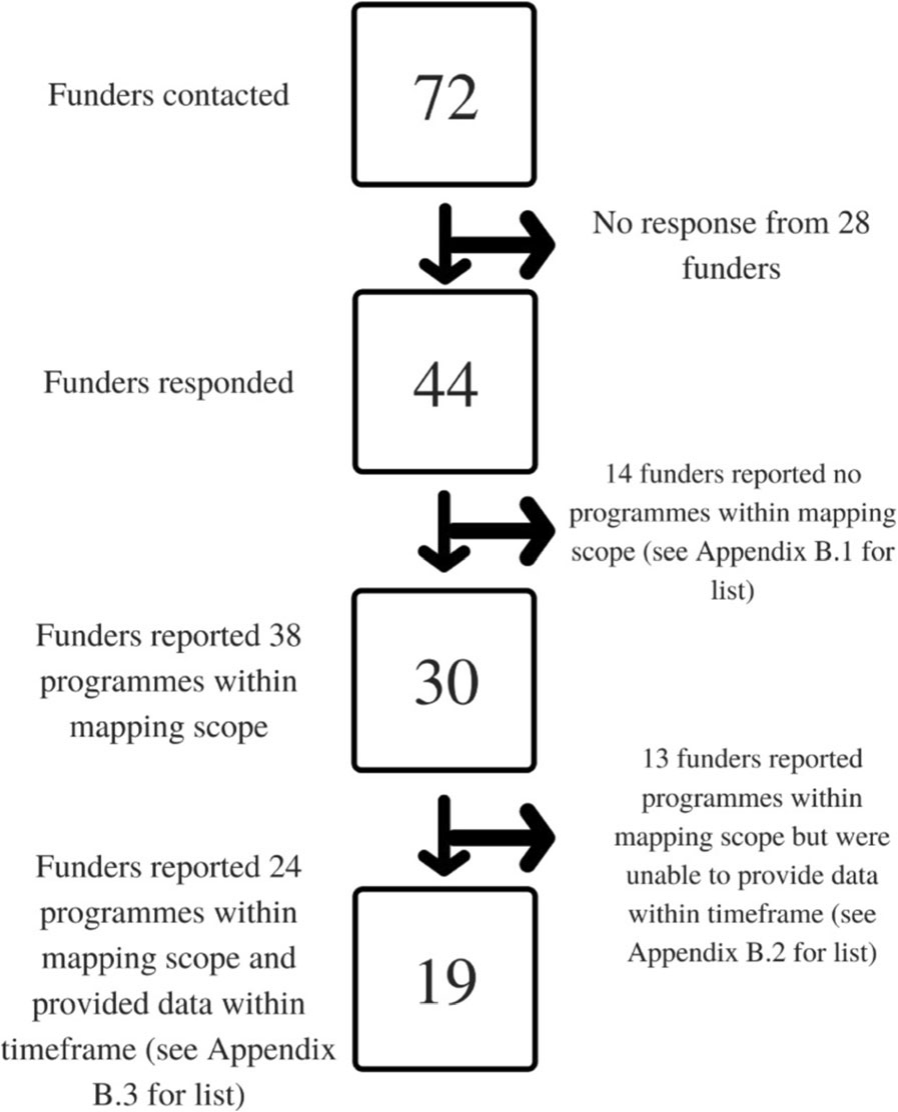
Flow chart showing number of funders at different stages of the mapping process. Note on Fig. 1: Because Carnegie Corporation (Carnegie) and the International Development Research Centre (IDRC) each reported two relevant programmes but could only provide data on one, they are counted as “Funders that reported programmes within mapping scope and provided data within timeframe” and “Funders that reported programmes within mapping scope but were unable to provide data within timeframe”

Of the 72 external funders contacted, 44 responded (Fig. 1). Nearly all the funding organisations are in countries in Europe and North America. Five funding organisations are in sub-Saharan Africa, of which three were unable to provide data (the African Network for Scientific and Technological Institutions, Kenya; the Graca Machel Trust, South Africa; and the New Partnership for Africa’s Development, as a joint funder with The UK Department for International Development and the Wellcome Trust) and two were able to provide data (the African Population and Health Research Centre, Kenya, and the Mandela Rhodes Foundation, South Africa).

This mapping assessed data on 24 postgraduate programmes run by 19 funders. If a funder reported on more than one programme, data was pooled and considered collectively as that funder’s contribution. Note that contributions from Medical Research Council (MRC) Uganda Unit and MRC Unit in the Gambia were classified together under MRC UK. (See Additional file 1: Appendix B for details of funders and programmes, and Additional file 1: Appendix C for details of the Master’s and PhD grantees they support).

#### Analysis by funder

**Fig. 2:**
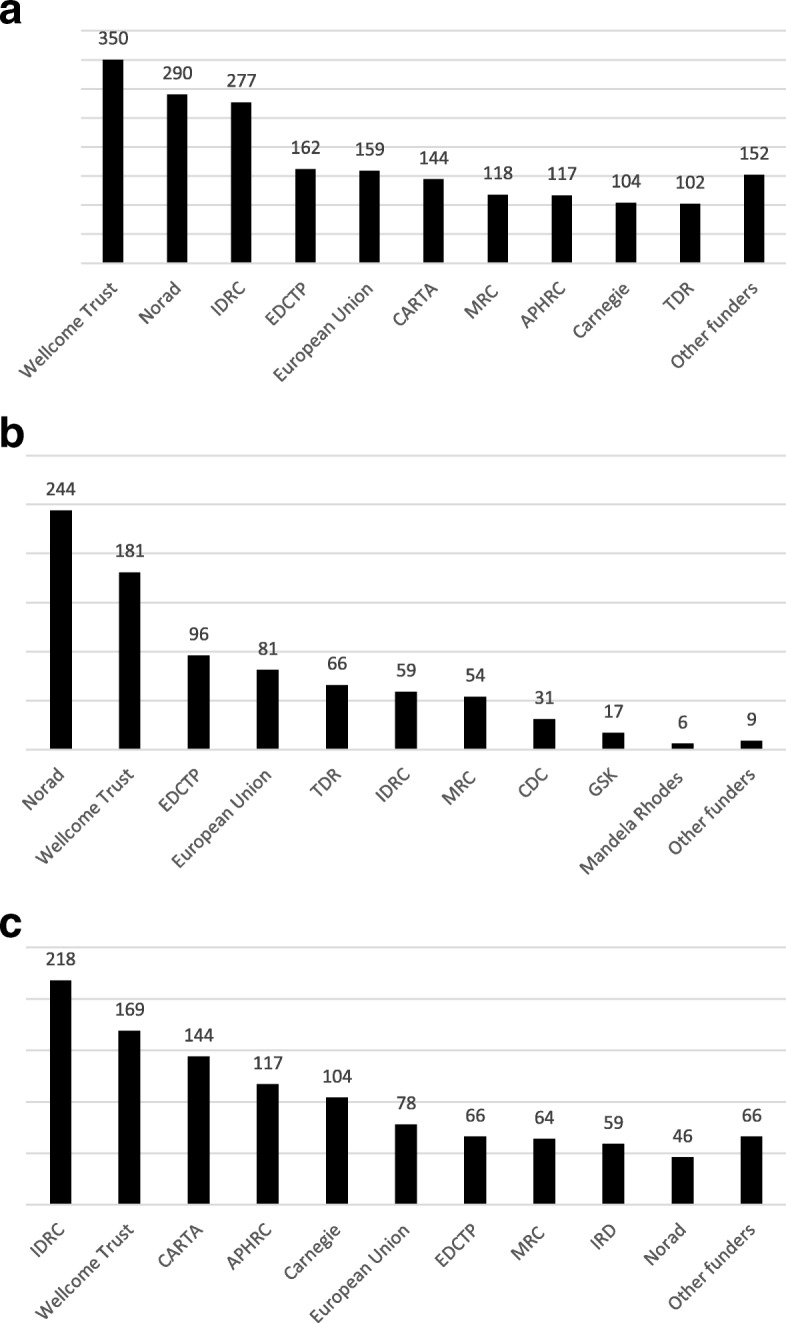
Number of postgraduate grantees supported by external funders, 2012–2017: (**a**) all, (**b**) Master’s, and (**c**) PhD. **a** New abbreviations used above include Norwegian Agency for Development Cooperation (NORAD), European & Developing Countries Clinical Trials Partnership (EDCTP), Consortium for Advanced Research Training in Africa (CARTA), African Population Health Research Centre (APHRC). “Other funders” include Institut de recherche pour le développement (IRD), Centre for Disease Control (CDC), German Academic Exchange Service (DAAD), GlaxoSmithKline (GSK), Mandela Rhodes Foundation, Beit Trust, Commonwealth Scholarships, Swedish International Development Cooperation Agency (Sida), and Institut Pasteur. **b** “Other funders” include Commonwealth Scholarships, Beit Trust and Sida. **c** “Other funders” include TDR, DAAD, GSK, Beit Trust and Institut Pasteur

Overall, support for 1975 postgraduate grantees was reported. This includes 844 Master’s grantees and 1131 PhD grantees. Figure 2 shows the number of postgraduate grantees supported by each funder, for all grantees (Fig. 2a), Master’s grantees (Fig. 2b), and PhD grantees (Fig. 2c).

#### Characteristics of grantees:

##### Country of origin

**Fig. 3:**
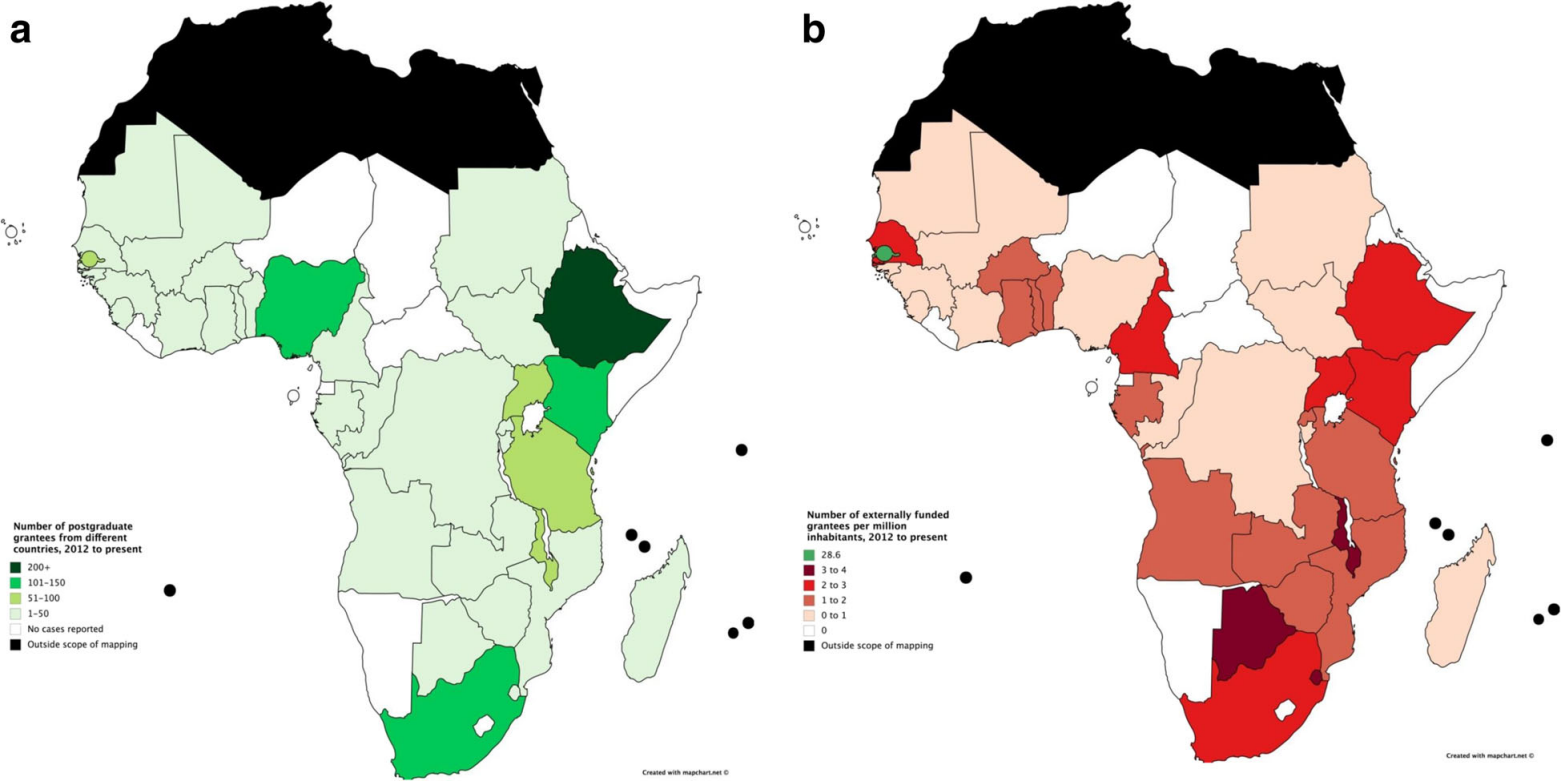
**a** Number of grantees by country of origin, 2012–2017. **b** Number of grantees by country of origin per million inhabitants per country, 2012–2017

Of the total 1975 grantees, information on country of origin was available for 1248 (63%). Figure 3(a) shows the number of grantees by country of origin, 2012–2017, and Fig. 3(b) shows the number of grantees by country of origin per million inhabitants per country, 2012–2017, calculated using 2016 population data [13]. All countries had between 0 and 4 grantees per million inhabitants apart from the Gambia, with 28.6 grantees per million inhabitants. (See Additional file 1: Appendix C.2 for number and density of grantees by country of origin).

##### Gender

Among a total of 1975 grantees (Master’s and PhD), 655 (33%) were women, 770 (39%) were men, and gender information was not provided for 550 (28%).

##### Research area

Table 3 shows the number and percent of Master’s and PhD grantees respectively in nine categories of health research activity.

**Table 3:** Number and percent of Master’s and PhD grantees by category of health research activity

Category of health research activity	Master’s		PhD	
	n	%	n	%
Basic science	124	15	162	14
R&D	60	7	85	8
Implementation	53	7	70	6
Social science	87	10	169	15
Health policy	0	0	32	2.8
Epidemiology	88	10	147	13
Health systems	15	1.8	199	18
Health technology	3	0.4	13	1.1
Health economics	4	0.5	4	0.4
Unreported	410	49	250	22
Total	844	100	1131	100

Among 844 Master’s and 1131 PhD grantees, information was available on both gender and research category for 619 Master’s (73.3%) and 806 PhD grantees (71.3%). Table 4 shows the gender breakdown among Master’s and PhD grantees in nine categories of health research activity. Among Master’s grantees, health systems and social sciences were more common research areas for women than men, while basic science and R&D were more common among men than women. Similarly, more men PhD grantees studied basic science, R&D and epidemiology than women grantees.

**Table 4:** Gender breakdown among Master’s and PhD grantees in nine categories of health research activity

Category of health research activity	Master’s			PhD		
	Female	Male	% Female	Female	Male	% Female
Basic science	13	26	33	31	52	37
R&D	15	21	42	26	38	41
Implementation	19	28	40	28	25	53
Social science	14	5	74	74	73	50
Health policy	0	0	–	12	16	43
Epidemiology	9	13	41	28	42	40
Health systems	11	3	79	63	80	44
Health technology	0	2	0	3	8	27
Health economics	0	0	–	2	1	67
Unreported	16	20	44	6	21	22
Total	97	118	45	273	356	43

##### Characteristics of institutions

**Fig. 4:**
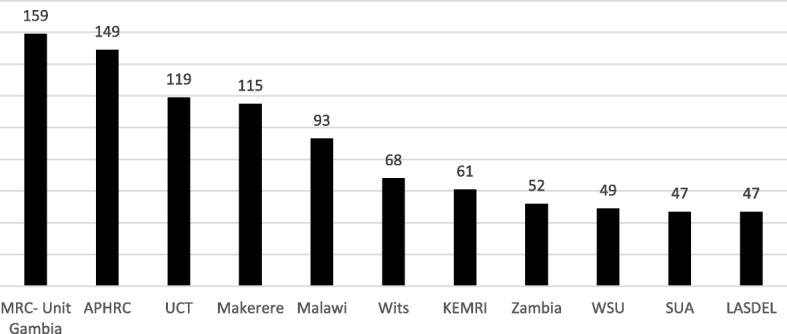
Number of externally funded postgraduate grantees hosted by the top institutions (ranked by number of grantees), 2012–2017

Institution of study was reported for 1940 of 1975 grantees, which totalled 133 institutions in the region. Of these, 22 institutions only hosted externally funded Master’s; 61 only hosted externally funded PhDs. Figure 4 shows the number of externally funded grantees at the eleven institutions which collectively hosted 49.4% of grantees (*n* = 959). (See Additional file 1: Appendix C.4 for a figure showing the number of externally funded Master’s grantees hosted for institutions with top 10 greatest numbers of Master’s grantees; see Additional file 1: Appendix C.5 for a figure showing the number of externally funded Master’s grantees hosted for institutions with top 10 greatest numbers of PhD grantees; see Additional file 1: Appendix C.5 for a figure showing the number of externally funded PhD grantees at the top eleven institutions. See Additional file 1: Appendix D for a legend of abbreviations used in Fig. 4 and in Additional file 1: Appendices C.4 and C.5). The MRC Unit in the Gambia, Makerere University, the University of Malawi and the Kenya Medical Research Institute all make notable contributions to hosting both Master’s and PhD grantees. Otherwise, there is considerable variation between institutions’ support for these grantees.

**Fig. 5:**
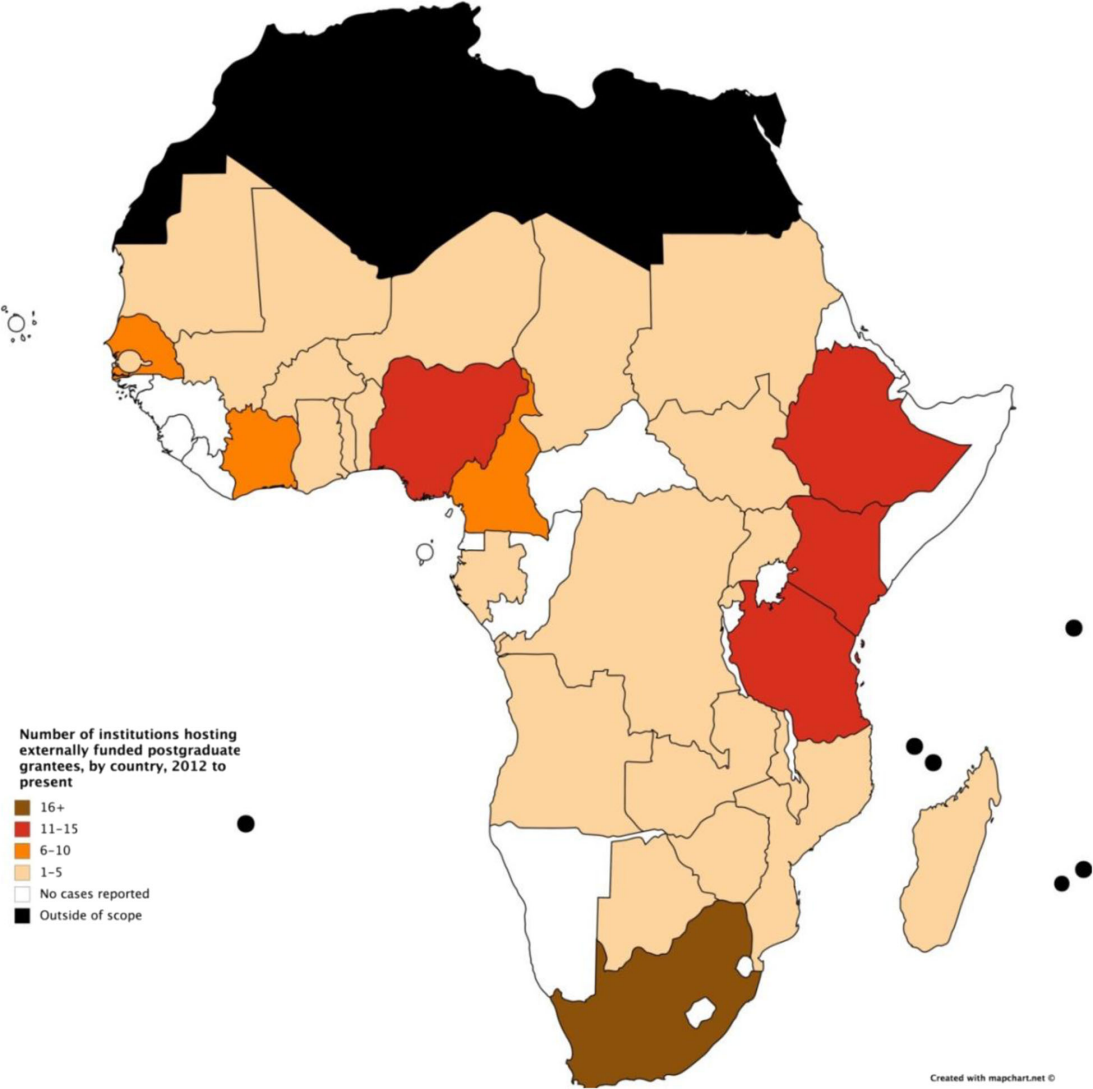
Number of institutions (in 5 groups of numbers) receiving external support for postgraduate grantees by country, 2012–2017

Grantees were reported to study at 133 institutions across 30 countries. Figure 5 shows the number of institutions hosting externally funded postgraduate grantees in countries in sub-Saharan Africa, in terms of number of institutions, divided into five groups. (See Additional file 1: Appendix E for a figure showing the absolute number of institutions hosting externally funded postgraduate grantees in sub-Saharan Africa).

**Fig. 6:**
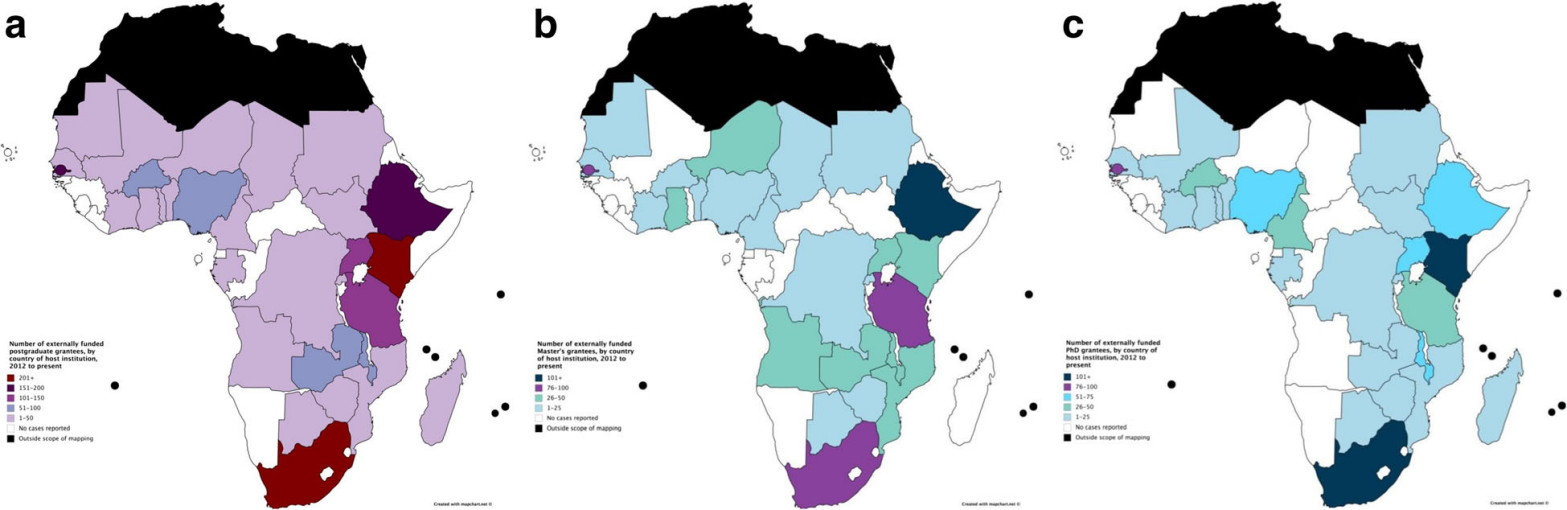
**a** Number of externally funded postgraduate grantees by country of host institution, 2012–2017. **b** Number of externally funded Master’s grantees by country of host institution, 2012–2017. **c** Number of externally funded PhD grantees by country of host institution, 2012–2017

Figure 6 shows the number of grantees hosted at institutions within each country, for (a) all grantees, (b) Master’s grantees, and (c) PhD grantees. Some countries only hosted either Master’s or PhD but not both. Nearly half of postgraduate grantees were reported to study in four countries: Ethiopia (*n* = 190), Kenya (*n* = 302), South Africa (*n* = 345) and the Gambia (*n* = 159).

## Discussion

In mapping externally funded training of postgraduate students in health research at institutions in sub-Saharan Africa, we identified 30 external funders, of whom 19 provided relevant data. The funders supporting the greatest numbers of Master’s and PhD grantees in the region were IDRC, NORAD and the Wellcome Trust. Our conclusions were made based on information provided from external funders, bearing in mind the limitation that the information was not always presented consistently across organizations.

Our results concern two aspects of geographic distribution of funders’ support in sub-Saharan Africa: where grantees come from (their country of origin) and where grantees go to (their institution of study). In both aspects, we found that funder support was strongest in eastern and southern Africa, and concentrated in six countries: Ethiopia, Kenya, Nigeria, South Africa, Tanzania and Uganda (see Figs. 3(a), (b), 4, 5 and 6). Apart from Nigeria, these countries have previously been classified as having established research capacity, and shown to attract the highest international research investments [4, 14]. Therefore, we found external support for postgraduate students to be geographically concentrated in countries with developed capacity. Botswana, Malawi and Swaziland were found to have the most concentrated support due to their relatively small populations. The high density of researchers coming from the Gambia, as shown in Fig. 3(b), can be explained by the strong external funding contributions for postgraduate training at MRC Unit the Gambia.

In general, external funders reported less support for western and central African nations and a series of countries on the eastern coast. Cape Verde, the Central African Republic, Djibouti, Equatorial Guinea, Eritrea, Lesotho, Liberia, Namibia, Sao Tome and Principe and Somalia were not found to have grantees coming or going. This reflects the low Human Development Index values of these countries, since R&D intensity is typically a reliable indicator of economic development [15]. Research funders have consistently avoided these countries for reasons such as corruption, inadequate infrastructure and political instability [14].

Furthermore, there was a strong concentration of support among select institutions (see Fig. 4). These institutions act as specialised research and training centres for the region, and maximize donor support while retaining African scientists and enabling the local research environment [16]. Some funders have significant historic and cultural ties to particular institutions because of common language and pre-existing communication networks, which may partially account for this trend [14]. As well, the positive cycle of strength leading to strength has continued to bias funders’ investment choices towards better established institutions. However, during the mapping process, all institutions expressed their need and ambition for greater support. There are benefits of investment in health research capacity strengthening in countries [17]. While the health and development benefits of research are relevant for all countries, the current external funding approach results in support for a select few institutions in a limited range of countries.

Another key finding of this mapping exercise was variation in funder support by type of degree funded and research area. Since young scientists form the foundation of any successful research institution, it is important that there is sufficient support given at the earlier career stages; yet, there were fewer externally funded Master’s grantees than PhDs. Our results (see Table 3) show a concentration of support at the beginning (i.e. towards the basic science end) of the research spectrum. Functional health research capacity requires a foundation of human resources dispersed evenly along the health research pipeline to prevent bottlenecks and ultimately to enable health system improvements [18]. More support for research areas farther along the pipeline will enable translation of research discoveries into health system improvements.

### Study strengths

To our knowledge, this report is the first attempt to map externally funded health researchers undertaking postgraduate degrees at institutions in countries in sub-Saharan Africa. We successfully identified 38 programmes that provide external funds for postgraduate students to study health research at institutions in sub-Saharan Africa. Of these, we collected and analysed data on 24 programmes, representing a 63% response rate from data sources. As an initial step towards mapping the full picture of support for health researchers in the region, the high response rate indicates great potential for funders to build on this study in improving data collection, analysis and reporting.

### Study limitations

The main limitation of this mapping exercise is the incompleteness of the data, arising from difficulties in: a) identifying all the relevant funding organizations; and b) obtaining responses from those identified. There was particular difficulty identifying funders from within the region, and funders from non-English-speaking countries. Of the 72 organizations identified and contacted, 44 organizations responded. Some large funders, including for example the US NIH and the Bill and Melinda Gates Foundation, were unable to compile a list of relevant programmes, or provide the number of postgraduate health researchers they support.

Many funders do not indicate on their websites if their programmes support postgraduate students. In the absence of a formal network for sharing data between funders, we relied on ad hoc assistance from the responding organizations’ staff, whose time limitations often represented a considerable barrier to obtaining information. While 30 funders reported 38 programmes within the mapping scope, 14 funders were unable to provide the requested data, or could only do so in part. The inaccessibility of data on gender and country of origin was particularly surprising given the importance of such data in assessing equity. Data on research area was difficult to gather because funders tend to classify their contribution by disease research topic (e.g. HIV/AIDS).

There is a wide range of possible arrangements by which external funding supports postgraduate students at institutions in the region. In the scope of this mapping, the institution of study referred to the institution which provided the main base and location of study and where the student was mainly funded. The institution of study is often also the degree-granting institution, but in some cases students obtained their degree from a different institution, sometimes outside of the region. This mapping did not therefore include the possible situation whereby an institution in the region hosts the student but the main beneficiary of the funding for training is a UK, US or European postgraduate programme where the student is registered for their degree. However the mapping did include those students for whom the main base for postgraduate training is at the UK Medical Research Council overseas units in the Gambia and Uganda and the programmes in Kenya, Malawi and South Africa funded by the Wellcome Trust, and who registered for their degree with the Open University in the UK. In this case, the main external funding goes to the institution of study and a small amount of the funding covers the registration fee at the Open University.

A challenge in deciphering data arose from low consistency in data storage among funders, who were generally unable to provide data in a uniform format. Extra data management was therefore required to make the data compatible for analysis. In addition, many funders provided their grantees’ thesis titles as their research area, so this was open to the possibility of misclassification.

## Conclusions

Regarding external support for activities aimed at strengthening research capacity, funders should explore future directions in dialogue with stakeholders in the region, including postgraduate grantees, national ministries of health and education, local research institutions and healthcare providers. Data-driven programmes of support would enable greater coherence among funding activities and help to avoid overlap and promote synergy. Our main conclusions concern firstly, the need for comprehensive information with more transparent and standardized data, and secondly, recommendations for funders to consider to address the observed uneven distribution of support for international research training at institutions in sub-Saharan Africa (for example, regarding country of origin of researcher, location of institution supported, degree level, and field of research) (see Table 5).

**Table 5:** Summary of main recommendations for funders to consider regarding data and distribution of support

Data	
Build on this initial mapping of externally supported training of postgraduate students and on related initiatives such as the World RePORT to develop a complete mapping of all postgraduate health training for LMIC researchers. Use data to evaluate the scale and impact of all efforts to strengthen health research capacity in LMICs. Ensure that the minimum standardised data for collection, analysis and reporting includes the following: each grantee’s institution of study and degree-granting institution; breakdown of funding for the institution of study and for the degree-granting institution; country of origin and gender of the grantee; and the research area of study. Relate these efforts to factors such as population, national human development index and economic development.	
Distribution of support	
Consider the optimum distribution of support for the two approaches based on 1) competitive research excellence and 2) equitability, which would achieve the most widespread health and development gains. Consider the optimum distribution of support for researchers at different career stages and for research on different parts of the research spectrum, which would respectively maximise the contribution of research capacity to the development of national research systems and the translation of research discoveries into health system improvements.	

Firstly, concerning information needs, this initial mapping is a first step in providing data on the distribution of support for training postgraduate research students to inform the dialogue, and paves the way for funders to collaborate in developing an online database which is updated annually. This would complement related initiatives such as the World RePORT [12], where major funders of health research in LMICs share key information about their activities. The minimum standardised data for collection, analysis and reporting should include each grantee’s institution of study and degree-granting institution, breakdown of funding for the institution of study and for the degree-granting institution, country of origin and gender of the grantee, and the research area of study. Funders should explore how to build on this initial mapping of externally supported training of postgraduate students and develop a complete mapping of all postgraduate health training for LMIC researchers. This in turn can contribute to evaluating the scale and impact of all efforts to strengthen health research capacity in LMICs. Further analysis could relate these efforts to factors such as population, national human development index and economic development.

Secondly, concerning recommendations for funders to consider, the current external funding approach tends to focus support on institutions selected on the basis of competitive research excellence. This results mainly in support for the same limited range of institutions in the same limited range of countries. A wider range of institutions and countries tend not to receive support because they are not competitive, yet still could benefit from support for strengthening research capacity as part of the development of national research systems to enable them to obtain the health and development benefits of research in the medium and long term. Funders should consider the optimum distribution of support for the two approaches, based on competitive research excellence and on equitability, which would achieve the most widespread health and development gains.

The current external funding approach also tends to focus support more on PhD than on Masters training, and more on the upstream than on the downstream parts of the research spectrum. Funders should consider the optimum distribution of support for researchers at different career stages and for research on different parts of the research spectrum, which would respectively maximise the contribution of research capacity to the development of national research systems and the translation of research discoveries into health system improvements.

## Additional file

Additional file 1: Appendices A-C. (DOCX 76 kb)

## References

[1] Nuyens Y (2005). No development without research: a challenge for capacity strengthening.

[2] Building institutions through equitable partnerships in global health. London: Academy of Medical Sciences; 2012.

[3] Fellesson M. Research capacity in the new global development agenda: mobility, collaboration and scientific production among PhD graduates supported by Swedish development aid in Africa. Stockholm: expert Group for aid. Studies. 2017;

[4] Uthman OA, Wiysonge CS, Ota MO, Nicol M, Hussey GD, Ndumbe PM, et al. Increasing the value of health research in the WHO African region beyond 2015 – reflecting on the past, celebrating the present and building the future: a bibiliometric analysis. BMJ Open 2015; 5(3):e006340. doi: https://doi.org/10.1136/bmjopen-2014-006340 PMID: 25770227. 10.1136/bmjopen-2014-006340 PMC4360830 25770227

[5] Cardoso A, Breugelmans G, Manville C, Chataway J, Cochrane G, Snodgrass J (2014). Africa mapping: current state of health research on poverty-related and neglected infectious diseases in sub-Saharan Africa. The Hague.

[6] IJsselmuiden C, Marais DL, Becerra-Posada F, Ghannem H (2012). Africa's neglected area of human resources for health research - the way forward. S Afr Med J.

[7] Dean L, Gregorius S, Bates I, Pulford J (2017). Advancing the science of health research capacity strengthening in low-income and middle-income countries: a scoping review of the published literature, 2000–2016. BMJ Open.

[8] Regional overview: sub-Saharan Africa: UNESCO science report, towards 2030. Paris: United Nations Educational, Scientific and Cultural Organization; 2015. Available from: https://en.unesco.org/unesco_science_report/africa [cited 2017 July 21].

[9] Enoch J. Health research capacity strengthening: a UKCDS mapping. London: UK collaborative on development. Sciences. 2014;

[10] Thornton I (2015). Health research capacity strengthening: 2015 update to the UKCDS mapping.

[11] Mapping of health System Research institutions in eastern and southern Africa Amsterdam: Royal Tropical Institute; 2017.

[12] World RePORT https://worldreport.nih.gov/app/#!/

[13] UNICEF data: monitoring the situation on women and children [Internet]. New York: The United Nations Children's Fund; 2016. Available from: https://data.unicef.org/ [cited 2017 July 26].

[14] Fitchett JR, Head MG, Atun R. Infectious disease research investments follow colonial ties: questionable ethics. International health 2014; 6(1):74–76. doi: https://doi.org/10.1093/inthealth/iht036 PMID: 24464047. 10.1093/inthealth/iht036 24464047

[15] Human development report 2016. Human development for everyone. New York: United Nations Development Programme. p. 2016.

[16] Nyirenda T. Regional Networks of Excellence for Clinical Trials Proceedings of the EDCTP Stakeholders’ Meeting; 2007 May 8; Doula, Cameroon. European & Developing Countries Clinical Trials Partnership; 2007.

[17] Head MG, Goss S., Gelister Y, Alegana V, Brown RJ, Clarke SC, et al. Global funding trends for malaria research in sub-Saharan Africa: a systemic analysis. Lancet Glob Health 2017;5(8)e772-ee81. doi: 10.1016/S2214-109X(17)30245-0 PMID: 28668230. 10.1016/S2214-109X(17)30245-0 PMC5567191 28668230

[18] Fitchett JR, Fan Li J, Atun R. Innovative financing for late-stage global health research and development: the global health investment fund. International Health 2015; 8(1):3–4. doi: 10.1093/inthealth/ihv067 PMID: 26612852. 10.1093/inthealth/ihv067 26612852

