# Supplementary material for: Strengthening health research capacity in sub-Saharan Africa: mapping the 2012–2017 landscape of externally funded international postgraduate training at institutions in the region
Source: Global Health. 2018 Jul 31;14:77. doi: 10.1186/s12992-018-0395-0 (PMC6066939; doi:10.1186/s12992-018-0395-0)
Supplement: Supplementary file 1 — Appendices A-C. (DOCX 76 kb) [file 12992_2018_395_MOESM1_ESM.docx]

# Appendix A

Angola

Benin

Botswana

Burkina Faso

Burundi

Cameroon

Cape Verde

The Central African Republic (CAR)

Chad

Congo, The Republic of (Congo)

Congo, The Democratic Republic of (DRC)

Cote d’Ivoire

Djibouti

Equatorial Guinea

Eritrea

Ethiopia

Gabon

Gambia, the

Ghana,

Guinea

Guinea-Bissau

Kenya

Lesotho

Liberia

Madagascar

Malawi

Mali

Mauritania

Mozambique

Namibia

Niger

Nigeria

Rwanda

Sao Tome and Principe

Senegal

Sierra Leone

Somalia

South Africa

South Sudan

Sudan

Swaziland

Tanzania

Togo

Uganda

Zambia

Zimbabwe

List of African countries excluded:

Algeria

Comoros

Egypt

Libya

Mauritius

Morocco

Seychelles

Tunisia

# Appendix B.1

- Alliance for Health Systems and Policy Research (AHSPR), Switzerland
- Canadian Institute of Health Research (CIHR), Canada
- Canadian International Development Agency (CIDA), Canada
- The Christian Medical Association of Malawi (CHAM), Malawi
- Council on Health Research for Development (COHRED), Switzerland
- French Agency for Research on AIDS and Viral Hepatitis (ANRS), France
- Ford Foundation, United States of America
- Gesellschaft für Internationale Zusammenarbeit (GIZ), Germany
- Institut national de la santé et de la recherché médicale (INSERM), France
- Japan International Cooperation Agency (JICA), Japan
- National Research Foundation (NRF), South Africa
- OPEC Fund for International Development (OFID), Austria
- South Africa Medical Research Council (SAMRC), South Africa
- Spanish Agency for International Development Cooperation (AECID), Spain

# Appendix B.2

| Funder name | Programme name | Notes | Master’s grantees, PhD grantees, or both |
| --- | --- | --- | --- |
| Aga Khan Development Network (AKDN), Switzerland^[[1]](#footnote-1)^ | The International Scholarship Programme | Supports Kenyans, Tanzanians, Ugandans, Malagasy and Mozambicans | Both |
| African Network for Scientific and Technological Institutions (ANSTI), Kenya^[[2]](#footnote-2)^ | DAAD Postgraduate Training Fellowship | Awarded yearly for training in STEM | Both |
| Carnegie Corporation, United States of America^[[3]](#footnote-3)^ | Regional Initiative in Science and Education (RISE) | Established in 2008 to support university-based, research and teaching networks | PhD |
| The Department for International Development (DFID), the New Partnership for Africa’s Development (NEPAD) and the Wellcome Trust | Developing Excellence in Leadership Training and Science Africa (DELTAS)^[[4]](#footnote-4)^ | Administered through the Alliance for Accelerating Excellence in Science in Africa (AESA) ^[[5]](#footnote-5)^  11 DELTAS consortia spread across research institutions in sub-Saharan Africa | Both |
| Graca Machel Trust, South Africa^[[6]](#footnote-6)^ | Graca Machel Scholarship Programme | Scholarships for southern African women  Grantees may elect to study health research | Both |
| International Development Research Centre (IDRC), Canada^[[7]](#footnote-7)^ | Food, Environment and Health program | Aims to improve health and build healthier food systems  Prioritizes the prevention of food-related non-communicable diseases | Both |
| Margaret McNamara Education Grant (MMEG), United States of America^[[8]](#footnote-8)^ | The African Program | Scholarships for women  Grantees may elect to study health research | Both |
| National Institute of Health (NIH), United States of America | Unknown | From World RePORT, NIH is known to indirectly support postgraduate grantees. | Unknown |
| NIH and the Wellcome Trust | Human Heredity and Health in Africa (H3Africa)^[[9]](#footnote-9)^ | Administered through the Alliance for Accelerating Excellence in Science in Africa (AESA) ^[[10]](#footnote-10)^  Seeks to build capacity in genomics research in Africa | Both |
| Newton Fund^[[11]](#footnote-11)^ | Unknown | Newton Fund does provide PhD placements in their research calls.  In the Newton Fund South Africa, there are five current health related programmes and most grantees are South African. | Both |
| Netherlands Organisation for Scientific Research (NWO), Netherlands^[[12]](#footnote-12)^ | NWO-WOTRO Science for Global Development programmes | Global Health Policy and Health Systems research programme, finishing in 2017 | Both |
| Schlumberger Foundation, United States of America^[[13]](#footnote-13)^ | Faculty for the Future programme | Scholarships for women in STEM | Both |
| Special Programme of Research, Development and Research Training in Human Reproduction (HRP), Switzerland^[[14]](#footnote-14)^ | Unknown | Supports individuals from LMICs to develop a critical mass of researchers in sexual and reproductive health | Unknown |
| World Bank, United States of America^[[15]](#footnote-15)^ | The First Phase of the African Centres of Excellence (ACE1) | Finances competitively-chosen institutions in the region  Nine institutions specializing in health, located in Ghana, Nigeria, Ethiopia, Malawi, Tanzania, Uganda and Zambia | Unknown |

# Appendix B.3

| Funder name | Programme name | Details | Master’s grantees, PhD grantees, or both |
| --- | --- | --- | --- |
| African Population and Health Research Centre (APHRC), Kenya^[[16]](#footnote-16)^ | The African Doctoral Dissertation Research Fellowship (ADDRF) | Doctoral and postdoctoral fellowships jointly supported by APHRC and IDRC | PhD |
| Association for Commonwealth Universities, United Kingdom^[[17]](#footnote-17)^ | Commonwealth Scholarships in low and middle income countries | Researchers from Commonwealth LMICs are eligible  Grantees may elect to study health research | Master’s |
| Beit Trust, United Kingdom^[[18]](#footnote-18)^ | Beit Trust Postgraduate Scholarships | Researchers from Malawi, Zambia or Zimbabwe  Institutions in the UK or South Africa  Grantees may elect to study health research | Both |
| Carnegie Corporation, United States of America^[[19]](#footnote-19)^ | Bilateral partnerships | Partnerships with the University of Cape Town and the University of the Witwatersrand | PhD |
| Carnegie Corporation, the British Council, the Swedish International Development Cooperation Agency, the MacArthur Foundation, the Wellcome Trust, the Ford Foundation, the University of Gothenburg, and Google | Consortium for Advanced Research Training in Africa (CARTA), Kenya^[[20]](#footnote-20)^ | Administered through the APHRC  Seven cohorts of fellows since 2008 | PhD |
| Centre for Disease Control (CDC), United States of America^[[21]](#footnote-21)^ | Bilateral partnership | Partnership with the Faculty of Medicine at Universite Agohstino Neto (ended 2015)  10 researchers funded per year for Master’s in Public Health or Field Epidemiology and Laboratorial Training | Master’s |
| European Union, Belgium^[[22]](#footnote-22),^^[[23]](#footnote-23)^ | 7^th^ Framework Programme for Research and Technological Development (FP7) | Active 2007 to 2013  Grants research projects in Europe and beyond | Both |
|  | Horizon 2020 | Active 2014 to 2020  Grants research projects in Europe and beyond | Both |
| European & Developing Countries Clinical Trials Partnership (EDCTP), Netherlands^[[24]](#footnote-24)^ | EDCTP1 | Active 2003 to 2013  Funded through the European Commission’s FP6 programme  Funds clinical trials, capacity building and networking projects in Europe and Africa  The EU provided around half of the budget for EDCTP1 | Both |
| German Academic Exchange Service (DAAD), Germany^[[25]](#footnote-25)^ | Unknown | The DAAD Regional Office for Africa funds a series of health postgraduate programmes for sub-Saharan Africans at institutions in the region | Master’s |
| GlaxoSmithKline (GSK), United Kingdom^[[26]](#footnote-26)^ | Africa Academic Investment programme | Structured within GSK’s Global Health Programmes division | Both |
| Institut Pasteur, France^[[27]](#footnote-27)^ | Calmette & Yersin programme | Provides funding for the completion of a doctoral thesis within the Institut Pasteur International Network  Grantee may focus on infectious diseases or public health | PhD |
| International Development Research Centre (IDRC), Canada^[[28]](#footnote-28)^ | Maternal and Child Health programme^[[29]](#footnote-29)^ | Projects are related to health information systems and adolescent sexual and reproductive health and rights Focus is in West Africa, and the Middle East and North Africa regions | Both |
| L'Institut de Recherche pour le Développement (IRD), France^[[30]](#footnote-30)^ | Allocations de Rercherche pour une Thèse au Sud (ARTS) | Aims to strengthen health research capacity in LMICs | PhD |
| Mandela Rhodes Foundation (MRF), South Africa^[[31]](#footnote-31)^ | MRF Scholarship Programme | Scholarship and leadership development program for studies at institutions in South Africa  Grantees may elect to study health research | Master’s |
| Medical Research Council (MRC UK), United Kingdom | MRC Unit, the Gambia^[[32]](#footnote-32)^ | Aims to build critical mass in sub-Saharan African health research | Both |
|  | MRC Uganda^[[33]](#footnote-33)^ | Aims to build critical mass in sub-Saharan African health research | PhD |
| Norwegian Agency for Development Cooperation (Norad), Norway^[[34]](#footnote-34)^ | Norwegian Programme for Capacity Development in Higher Education and Research for Development (NORHED)^[[35]](#footnote-35)^ | Aims to strengthen the capacity of LMIC higher education institutions to be able to educate more and better graduates and to produce more and better research (by own researchers)  Ten programmes run through the Section for Research, Innovation and Higher Education at the Department for Climate, Energy, Environment and Research | Both |
| Special Programme for Research and Training in Tropical Diseases (TDR), Switzerland | TDR Postgraduate Training Scheme^[[36]](#footnote-36)^ | Postgraduate degrees in implementation research at seven institutions worldwide | Both |
|  | Population Health Vulnerabilities to Vector Borne Diseases programme^[[37]](#footnote-37)^ | Joint IDRC and TDR initiative  Supports five projects on health, vectors and climate change in the region | Both |
| Swedish International Development Cooperation Agency (Sida), Sweden^[[38]](#footnote-38)^ | Research Cooperation programme | Bilateral support for institutions in LMICs  Direct support for postgraduate degrees at the University of Rwanda, Universidad Eduardo Mondlane, Makerere University, and Muhimbili University for Health and Allied Sciences | Master’s |
| Wellcome Trust, United Kingdom^[[39]](#footnote-39)^ | Master’s and Training Fellowships in Public Health and Tropical Medicine programmes | Support for LMIC researchers doing postgraduate studies  Master’s programme comprises a 12-month taught course, plus an 18-month research project^[[40]](#footnote-40)^ | Both |
|  | Africa and Asia Programs (AAPs) | Three programmes in sub-Saharan Africa (as well as two in Asia): Africa Centre, at the Africa Centre for Population Health; The Malawi-Liverpool-Wellcome Trust Clinical Research Programme (MLW) at the University of Malawi; and the KEMRI-Wellcome Trust Research Programme (KWTRP) at KEMRI | PhD |
|  | African Institutions Initiative (AII) | Funds seven capacity-building consortia in health research in sub-Saharan Africa: CARTA,^[[41]](#footnote-41)^ AFRIQUEONE, IIDP, THRiVE, SACIDS, SACORE and SNOWS | PhD |

#

# Appendix C.1

| Organization | Direct or indirect | Number of Master’s grantees supported | Number of PhD grantees supported | Number of postgraduate grantees supported |
| --- | --- | --- | --- | --- |
| APHRC | direct | 0 | 117 | 117 |
| Beit Trust | direct | 3 | 2 | 5 |
| Carnegie | direct | 0 | 104 | 104 |
| CARTA | direct | 0 | 144 | 144 |
| CDC | indirect | 31 | 0 | 31 |
| Commonwealth Scholarships | direct | 4 | 0 | 4 |
| DAAD | unavailable | 0 | 25 | 25 |
| EC | indirect | 81 | 78 | 159 |
| EDCTP | indirect | 96 | 66 | 162 |
| GSK | direct | 17 | 2 | 19 |
| IDRC | Indirect | 59 | 218 | 277 |
| Institut Pasteur | direct | 0 | 1 | 1 |
| IRD | direct | 0 | 59 | 59 |
| Mandela Rhodes | direct | 6 | 0 | 6 |
| MRC | direct | 54 | 64 | 118 |
| Norad | direct | 244 | 46 | 290 |
| Sida | direct | 2 | 0 | 2 |
| TDR | both | 66 | 36 | 102 |
| Wellcome Trust | direct | 181 | 169 | 350 |

# Appendix C.2

| Country of origin | Number of grantees | Number of grantees per million inhabitants |  | Country of origin | Number of grantees | Number of grantees per million inhabitants |
| --- | --- | --- | --- | --- | --- | --- |
| Angola | 31 | 1.24 |  | Mali | 4 | 0.23 |
| Benin | 21 | 1.93 |  | Mauritania | 2 | 0.49 |
| Botswana | 8 | 3.54 |  | Mozambique | 38 | 1.36 |
| Burkina Faso | 22 | 1.22 |  | Nigeria | 108 | 0.59 |
| Burundi | 2 | 0.18 |  | Rwanda | 17 | 1.46 |
| Cameroon | 48 | 2.06 |  | Sénégal | 32 | 2.16 |
| Congo | 4 | 0.87 |  | Sierra Leone | 3 | 0.46 |
| Côte d'Ivoire | 17 | 0.75 |  | South Africa | 135 | 2.48 |
| DRC | 26 | 0.34 |  | South Sudan | 6 | 0.49 |
| Ethiopia | 206 | 2.07 |  | Sudan | 5 | 0.12 |
| Gabon | 3 | 1.74 |  | Swaziland | 4 | 3.11 |
| Gambia, the | 57 | 28.63 |  | Tanzania | 88 | 1.65 |
| Ghana | 28 | 1.02 |  | Togo | 9 | 1.32 |
| Guinea | 3 | 0.24 |  | Uganda | 87 | 2.23 |
| Guinea Bissau | 1 | 0.54 |  | Unspecified | 727 | NA |
| Kenya | 118 | 2.56 |  | Zambia | 21 | 1.30 |
| Madagascar | 2 | 0.08 |  | Zimbabwe | 24 | 1.54 |
| Malawi | 68 | 3.95 |  |  |  |  |

# Appendix C.3

|  | Number of Master’s grantees | Number of PhD grantees | Total number of postgraduate grantees |
| --- | --- | --- | --- |
| Woman | 294 (34.83%) | 361 (31.92%) | 655 (33.16%) |
| Man | 325 (38.51%) | 445 (39.35%) | 770 (38.99%) |
| Unspecified | 225 (26.66%) | 325 (28.74%) | 550 (27.85%) |
| Total | 844 (100%) | 1,131 (100%) | 1,975 (100%) |

# Appendix C.4

# Appendix C.5

# Appendix D

| Abbreviation | Institution name | Institution location |
| --- | --- | --- |
| APHRC | African Population and Health Research Centre | Kenya |
| Dilla | Dilla University | Ethiopia |
| Ibadan | University of Ibadan | Nigeria |
| KEMRI | Kenya Medical Research Institute | Kenya |
| LASDEL | Laboratoire d'Etude et de Recherche sur les Dynamiques Sociales et le Développement Local | Niger |
| Makerere | Makerere University | Uganda |
| Malawi | University of Malawi | Malawi |
| MRC Unit, Gambia | Medical Research Council Unit, the Gambia | the Gambia |
| SUA | Sokoine University of Agriculture | Tanzania |
| UAN | Agostinho Neto University | Angola |
| UCT | University of Cape Town | South Africa |
| UKZN | University of KwaZulu-Natal | Africa |
| UO | Universite de Ouagadougou | Burkina Faso |
| UWC | University of the Western Cape | South Africa |
| Wits | University of the Witwatersrand | South Africa |
| WSU | Wolaita Sodo University | Ethiopia |
| Zambia | University of Zambia | Zambia |

# Appendix E

1. (AGF, 2016) [↑](#footnote-ref-1)
2. (ANSTI, 2015) [↑](#footnote-ref-2)
3. (A Elwadie & A Johnson 2017, 12 July) [↑](#footnote-ref-3)
4. (AAS, 2017) [↑](#footnote-ref-4)
5. (A Neba 2017, 10 May) [↑](#footnote-ref-5)
6. (Graca Machel Trust, 2014) [↑](#footnote-ref-6)
7. (A Nayfeh 2017, 5 July) [↑](#footnote-ref-7)
8. (MMEG, 2015) [↑](#footnote-ref-8)
9. (M Skelton 2017, 11 July) [↑](#footnote-ref-9)
10. (A Neba 2017, 10 May) [↑](#footnote-ref-10)
11. (S Nderitu 2017, 23 May), (V Bungane 2017, 6 June) [↑](#footnote-ref-11)
12. (M Verschoor 2017, 29 May) [↑](#footnote-ref-12)
13. (Schlumberger Foundation, 2010) [↑](#footnote-ref-13)
14. (D Khan 2017, 13 June) [↑](#footnote-ref-14)
15. (H Bayusuf 2017, 27 June) [↑](#footnote-ref-15)
16. (P Ngure 2017, 14 June) [↑](#footnote-ref-16)
17. (The Association of Commonwealth Universities, 2017) [↑](#footnote-ref-17)
18. (W Barton 2017, 15 June) [↑](#footnote-ref-18)
19. (A Elwadie & A Johnson 2017, 12 July) [↑](#footnote-ref-19)
20. (P Ngure 2017, 14 June) [↑](#footnote-ref-20)
21. (E Catumbela 2017, 29 May) [↑](#footnote-ref-21)
22. (I Peñas Jiménez 2017, 22 June) [↑](#footnote-ref-22)
23. Data for both FP7 and Horizon 2020 was adapted from the responses of individual research teams. Total response rate of research teams was 23%. [↑](#footnote-ref-23)
24. (T Nyirenda 2017, 11 May) [↑](#footnote-ref-24)
25. (K Wambua 2017, 4 July) [↑](#footnote-ref-25)
26. (S Hassane 2017, 6 July) [↑](#footnote-ref-26)
27. (M Lucas-Hourani 2017, 26 June) [↑](#footnote-ref-27)
28. (A Nayfeh 2017, 5 July) [↑](#footnote-ref-28)
29. The IDRC did not always specify which type of postgraduate degree was funded, describing them instead as “Master’s/PhD degrees funded”; due to their research topics, these were all assumed to be PhDs. [↑](#footnote-ref-29)
30. (A Bricout 2017, 28 June) [↑](#footnote-ref-30)
31. (MRF, 2017) [↑](#footnote-ref-31)
32. (A Jaye & A Drammeh 2017, 29 June) [↑](#footnote-ref-32)
33. (A Elliot 2017, 29 June) [↑](#footnote-ref-33)
34. (S Breivik 2017, 15 June) [↑](#footnote-ref-34)
35. NOHRED data only included candidates already enrolled or graduated by 2016, as the only figures available for 2017 were target projections. [↑](#footnote-ref-35)
36. (E Kamau 2017, 8 May) [↑](#footnote-ref-36)
37. (B Ramirez 2017, 10 May) [↑](#footnote-ref-37)
38. (M Bejarano 2017, 31 May), (C de Carvalho 2017, 5 June) [↑](#footnote-ref-38)
39. (E Gillies, 16 June) [↑](#footnote-ref-39)
40. For the Training Fellowships in Public Health and Tropical Medicine, Wellcome Trust was unable to distinguish between Training Fellows in doctoral or postdoctoral positions, so these grantees were all logged as PhD grantees (n=26). [↑](#footnote-ref-40)
41. For the purpose of this report, information on CARTA was reported and analyzed separately from the other AIIs. It is presented individually. [↑](#footnote-ref-41)
